# Supplementary material for: Dual inhibition of the terminal oxidases eradicates antibiotic‐tolerant Mycobacterium tuberculosis
Source: EMBO Mol Med. 2020 Dec 7;13(1):e13207. doi: 10.15252/emmm.202013207 (PMC7799364; doi:10.15252/emmm.202013207)
Supplement: Supplementary file 1 — Appendix [file EMMM-13-e13207-s001.pdf]

Appendix

Dual inhibition of the terminal oxidases eradicates antibiotic-tolerant *Mycobacterium tuberculosis*

<u>Table of Contents</u>

Appendix Figures

Appendix Figure S1

Appendix Figure S2

Appendix Figure S3

Appendix Tables

Appendix Table S1

Appendix Table S2

Appendix Table S3

Appendix Table S4

Appendix Table S5

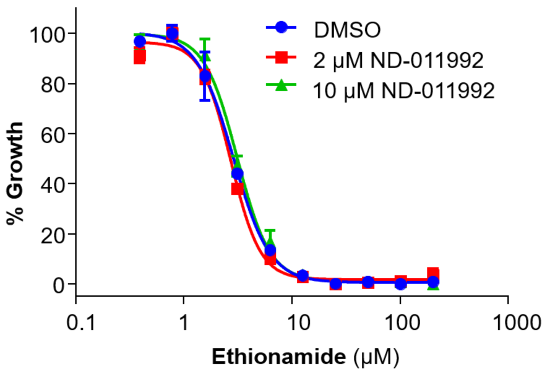

APPENDIX FIGURE S1. Effect of ND-011992 treatment in Ethionamide minimum inhibitory concentration (MIC). *M. tuberculosis* H37Rv cultures were treated with varying concentrations of ethionamide in the presence of DMSO (blue circles), 2 µM ND-011992 (red squares), or 10 µM ND-011992 (green triangles). Data are expressed as the mean ± S.D. of triplicates for each condition.

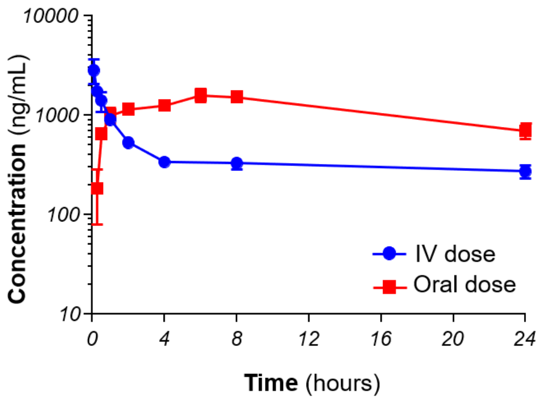

APPENDIX FIGURE S2. Plasma concentration of ND-011992 after intravenous and oral dosing in male Swiss albino mice. Plasma concentration of ND-011992 was quantified over a 24-hour period after an intravenous (IV) dose of 2 mg/kg (blue circles) or oral administration of 10 mg/kg (red squares).

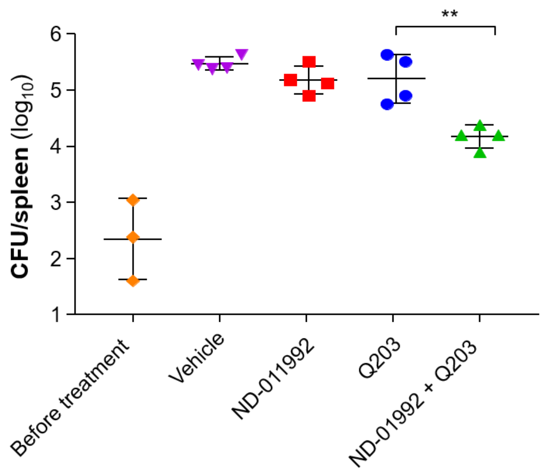

APPENDIX FIGURE S3. The drug combination ND-011992/Q203 reduces bacterial load in the spleen of *M. tuberculosis*-infected mice. Efficacy of ND-011992-Q203 combination treatment in a mouse model of acute tuberculosis. Bacterial load (CFU) were enumerated in the spleen of mice before treatment (orange diamonds), or after 5 daily oral administration of vehicle control (purple triangles), 5 mg/kg Q203 (blue circles), 25 mg/kg ND-011992 (red squares), or 25 mg/kg ND-011992 + 5 mg/kg Q203 (green triangles). **$P < 0.01$, unpaired Student's t-test, two-tailed; n = 4.

APPENDIX TABLE S1. Cyt-*bd* over-expression reduced the potency of ND-011992. *M. bovis* BCG (transformed with empty pMV262 vector or pMV262-*cydABDC*) strains were treated with a dose-range of ND-011992, Q203, or BDQ for 15 hours before quantification of intracellular ATP levels.

	ATP IC_{50} (µM)		
	ND-011992	Q203	BDQ
pMV262	0.52 ± 0.014	0.0010 ± 0.000045	0.16 ± 0.0069
pMV262-*cydABDC*	17.2 ± 3.64	0.0012 ± 0.000206	0.19 ± 0.014

APPENDIX TABLE S2. Minimum Inhibitory Concentration 50% (MIC_{50}) of ND-011992 in *M. tuberculosis* H37Rv ∆ctaE-qcrCAB.

MIC_{50} (μM)	Repeat 1		Repeat 2	
	**ND-011992**	**BDQ**	**ND-011992**	**BDQ**
**WT H37Rv**	25.2	1.02	25.5	0.50
**∆ctaE-qcrCAB**	0.76	0.56	0.23	0.33
**∆ctaE-qcrCAB::comp.**	3.54	0.63	20.0	0.65

APPENDIX TABLE S3. Frequency of spontaneous resistance to ND-011992 + Q203 in *M. tuberculosis* H37Rv and *M. bovis* BCG.

**Drug concentration** (μM)		**Frequency of Resistance**	
Q203	ND-011992	H37Rv	BCG
0.1	0	$2.4×10^{-8}$	$1.0×10^{-8}$
	6	$2.1×10^{-9}$	$6.6×10^{-9}$
	10	$< 2.3×10^{-10}$	$3.7×10^{-10}$
0.5	0	N.D.	$6.7×10^{-9}$
	6	N.D.	$7.3×10^{-10}$
	10	N.D.	$< 3.7×10^{-10}$

APPENDIX TABLE S4. *In vitro* pharmacokinetic and toxicity of ND-011992

**Metabolic stability**	Stability in mouse plasma (% remaining after 2h)			110.3% (Propantheline bromide: 12.1%)
	Stability in simulated gastric fluid (% remaining after 24h)			99.02% (omeprazole: 0.00%)
	Microsomal stability	Human	CL_{int}, µL min^{-1} mg^{-1}	15.7
			% remaining after 60 min	58.1% (Propafenone: 0.4%)
		Mouse	CL_{int}, µL min^{-1} mg^{-1}	<9.6
			% remaining after 60 min	87.6% (Propafenone: 0.2%)
**Drug permeability**	Caco2 assay (mean P_{app}, 10^{-6} cm s^{-1})			<0.48
**Cytotoxicity**	HepG2 (IC_{50})			>20 µM

APPENDIX TABLE S5. Pharmacokinetic properties of ND-011992 in mice after intravenous (IV) and oral (PO) administration

		IV	PO
Dose	mg/kg	2.00	10.00
C_{max}	ng/mL	3583.45	1601.80
T_{max}	H	-	6.00
V_d	L/kg	5.35	-
Cl	mL/min/kg	0.96	-
$T_{1/2}$	h	64.11	>18
$AUC_{0\text{-last}}$	ng· h/mL	9298.20	26954.80
$AUC_{0\text{-}\infty}$	ng· h/mL	34603.48	41694.93
AUC_{extra}	%	73.13	35.35
$MRT_{0\text{-last}}$	h	9.46	11.10
F	%	-	57.98
