# Supplementary material for: Dual inhibition of the terminal oxidases eradicates antibiotic‐tolerant Mycobacterium tuberculosis
Source: EMBO Mol Med. 2020 Dec 7;13(1):e13207. doi: 10.15252/emmm.202013207 (PMC7799364; doi:10.15252/emmm.202013207)
Supplement: Supplementary file 2 — Expanded View Figures PDF [file EMMM-13-e13207-s002.pdf]

Expanded View Figures

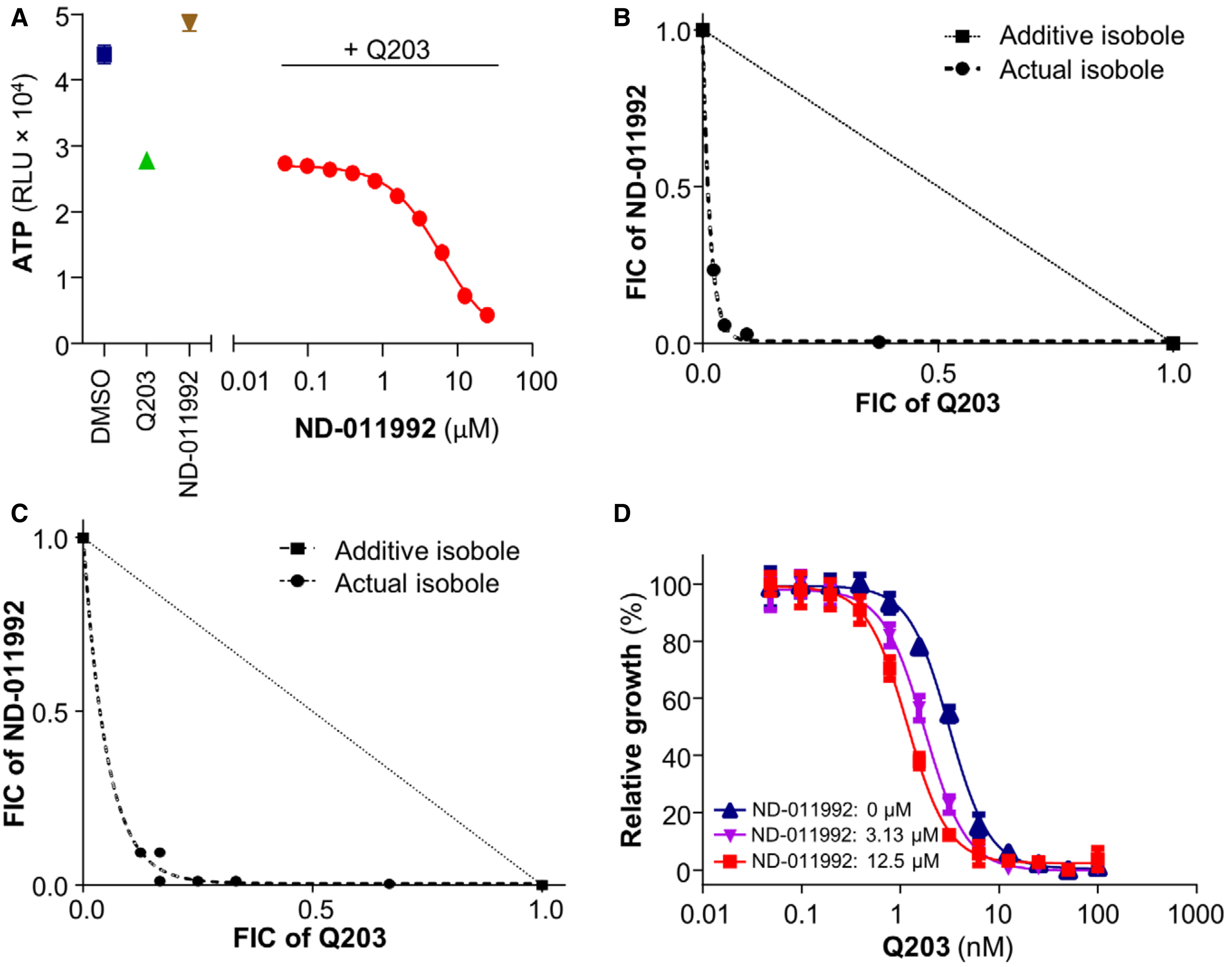

Figure EV1. ND-011992 synergizes with Q203 to inhibit ATP production and growth in mycobacteria.

A Effect of ND-011992 on the intracellular ATP level in *M. tuberculosis* H37Rv. The bacteria were treated with DMSO (blue square), 100 nM Q203 (green triangle), 25 μM ND-011992 (brown triangle), and varying concentrations of ND-011992 in the presence of 100 nM Q203 (red circles) for 15 h.

B Isobologram analysis reflecting the interactions between ND-011992 and Q203 in an intracellular ATP depletion checkerboard assay in *M. bovis* BCG.

C Isobologram analysis reflecting the interactions between ND-011992 and Q203 in an intracellular ATP depletion checkerboard assay in *M. tuberculosis* H37Rv.

D Effect of ND-011992 on the dose-dependent growth inhibition curve of Q203 in *M. tuberculosis* H37Rv. Blue triangles: Q203 alone; purple triangles: with 3.13 μM ND-011992; red squares: with 12.5 μM ND-011992. Data are expressed as the mean ± SD of triplicates for each condition.

© 2021 The Authors

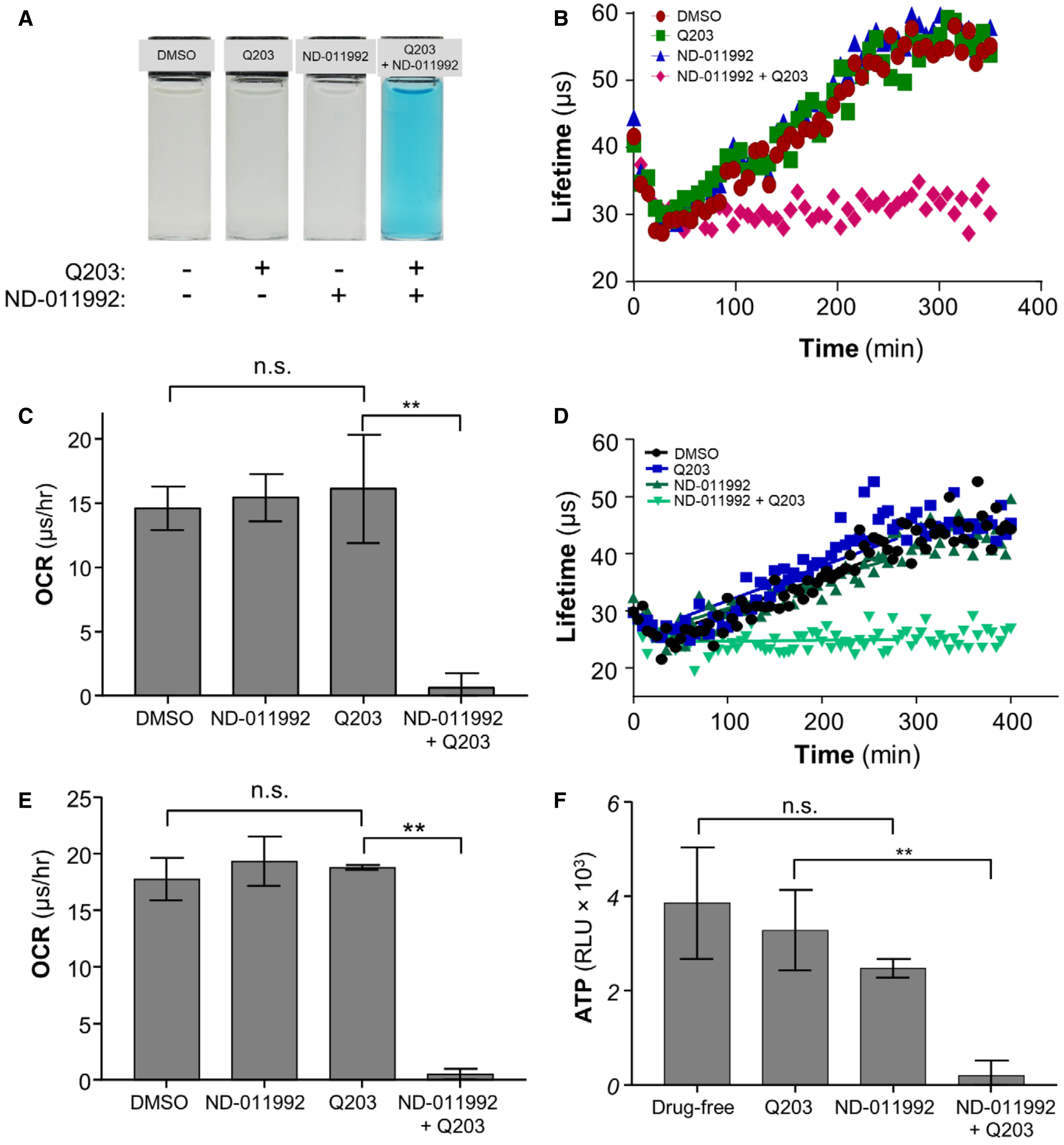

Figure EV2.

 © 2021 The Authors

Figure EV2. ND-011992-Q203 combination inhibits oxygen respiration in mycobacteria.

A Oxygen consumption assay in *M. tuberculosis* H37Rv using methylene blue as an oxygen sensor.
B Oxygen consumption assay in *M. tuberculosis* H37Rv using the MitoXpress Xtra® probe. H37Rv was incubated with the oxygen probe in the presence of 1% DMSO (red circles), 100 nM Q203 (green squares), 25 μM ND-011992 (blue triangles), or 25 μM ND-011992 + 100 nM Q203 (pink diamonds).
C Oxygen consumption rate of *M. tuberculosis* H37Rv derived from the MitoXpress Xtra® assay (panel B). **P = 0.0036, unpaired Student's t-test; two-tailed; n = 3.
D Oxygen consumption assay in *M. bovis* BCG using the MitoXpress Xtra® probe. *M. bovis* BCG was incubated with the oxygen probe in the presence of 1% DMSO (black circles), 100 nM Q203 (blue squares), 25 μM ND-011992 (dark green triangles), or 25 μM ND-011992 + 100 nM Q203 (light green triangles).
E Oxygen consumption rate of *M. tuberculosis* H37Rv derived from the MitoXpress Xtra® assay (panel D). **P = 0.00000042, unpaired Student's t-test; two-tailed; n = 3.
F Effect of ND-011992 on the ATP synthesis of *M. tuberculosis* H37Rv $mc^2$6230 inverted-membrane vesicles (IMV) using the Roche Bioluminescence Assay Kit CL II. Q203 was used at 100 nM, and ND-011992 was used at 40 μM. The figure shows averages and standard deviations of triplicate repeats with the blank subtracted from all conditions. P-value between drug-free and ND-011992: 0.1159; **P = 0.0020, unpaired Student's t-test; two-tailed; n = 3.

Data information: Data are expressed as the mean ± SD of triplicates for each condition.

© 2021 The Authors

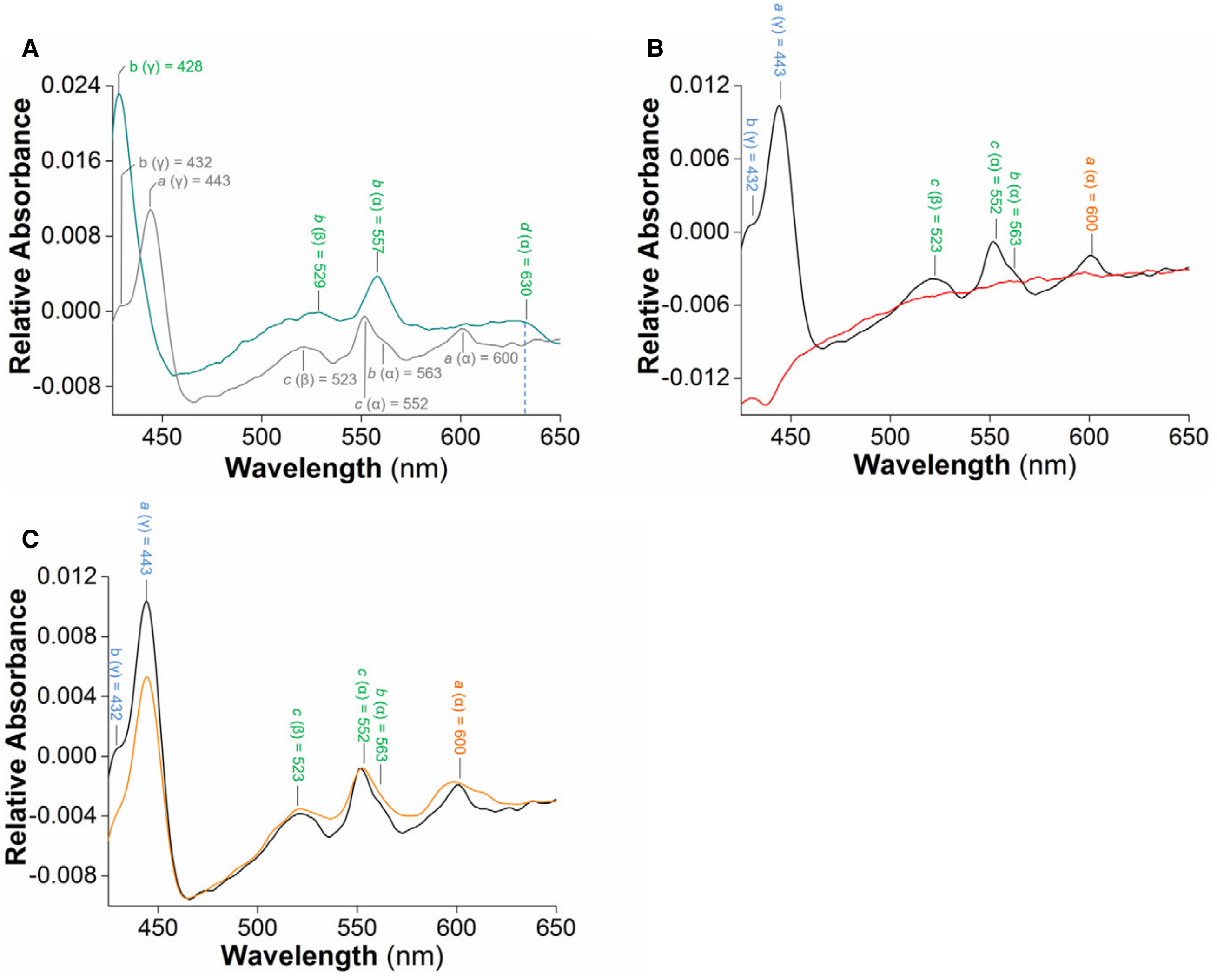

Figure EV3. ND-011992 does not affect electron transfer within the cytochrome *bcc:aa₃* supercomplex.

A NADH-driven electron transfer within plasma membrane vesicles (IMVs) of the wild-type (gray) and Δ*qcrCAB M. smegmatis (green)*. The difference spectrum of IMVs from WT *M. smegmatis* after addition of the electron donor NADH shows the major absorbance peaks for heme *a*, *b*, and *c* of the cytochrome *bcc-aa*$_3$ supercomplex. The characteristic peaks of Cyt-*bd* are displayed after reduction by 2 mM NADH in the Δ*qcrCAB* background.

B, C Effect of Q203 and ND-011992 on NADH-driven electron transfer in wild-type *M. smegmatis* IMVs. (B) The NADH-based difference spectra in the absence (black) and presence of 7.5 μM Q203 (red) reflect the effect of Q203 on the reduction of hemes in the cytochrome *bcc-aa*$_3$. (C) The NADH-based difference spectra in the absence (black) and the presence of 10 μM ND-011992 (orange).

 © 2021 The Authors

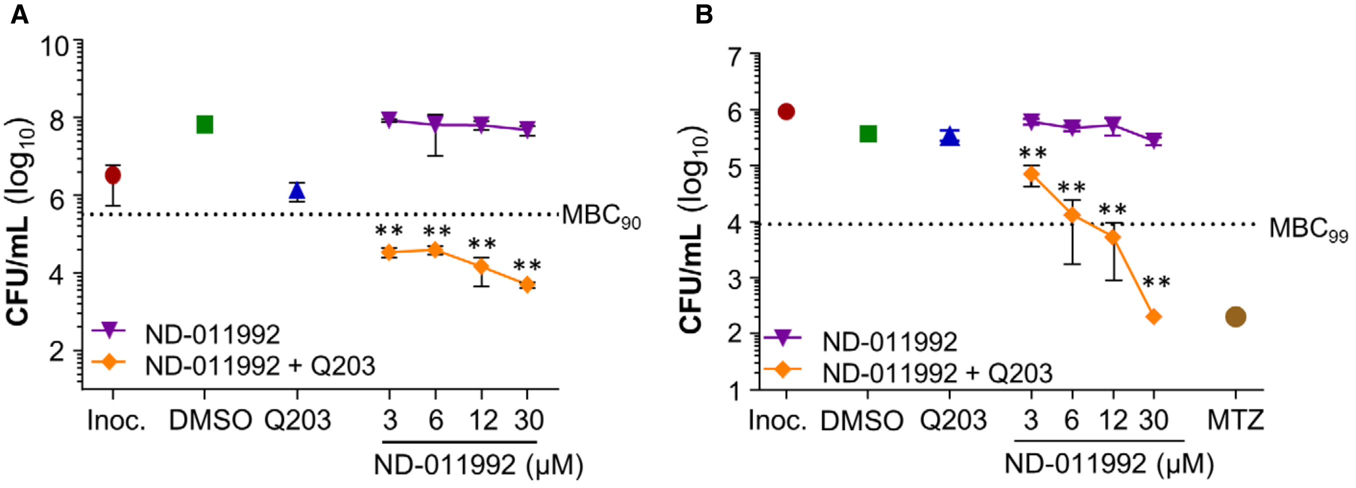

Figure EV4. The drug combination ND-011992-Q203 is bactericidal against *Mycobacterium bovis* BCG.

A Bactericidal activity of ND-011992 and ND-011992-Q203 combination in replicating *M. bovis* BCG. Q203 was used at 100 nM, and ND-011992 used at 3, 6, 12, and 30 μM alone or in combination with Q203. Dotted line represents 90% reduction in CFU/ml compared with the initial inoculum at time 0. Exact *P*-values (from left to right): 0.0002, 0.0002, 0.0003, 0.00002.

B Bactericidal activity of ND-011992 and ND-011992-Q203 combination in *M. bovis* BCG under oxygen-starved condition. Metronidazole (MTZ) was used at 200 μM, Q203 was used at 100 nM, and ND-011992 was used at 3, 6, 12, and 30 μM. Bacterial viability was determined via CFU enumeration after 10 days of treatment. Dotted line represents 99% reduction in CFU/mL compared with the initial inoculum at time 0. Exact *P*-values (from left to right): 0.0024, 0.0075, 0.0029, 0.0000003.

Data information: $^{**}P < 0.01$, unpaired Student's t-test, one-tailed; $n = 3$; comparing Q203 alone *vs* Q203 + ND-011992. Data are expressed as the mean ± SD of triplicates for each condition.

© 2021 The Authors
